# What is the impact of PPAR-γ agonist-rosiglitazone on ovarian reserve after hysterectomy? An experimental study

**DOI:** 10.3906/sag-2002-117

**Published:** 2020-08-26

**Authors:** Burcu GÜNDOĞDU, Ömer Lütfi TAPISIZ, Berna DİLBAZ, Serkan Barış MÜLAZIMOĞLU, Kamil Hakan MÜFTÜOĞLU, Betül DÜNDAR, Ümit GÖKTOLGA

**Affiliations:** 1 Department of Obstetrics and Gynecology, University of Health Sciences, Etlik Zübeyde Hanım Women’s Health Training and Research Hospital, Ankara Turkey; 2 Department of Gynecology and Obstetrics, Faculty of Veterinary Medicine, Ankara University, Ankara Turkey; 3 Department of Pathology, University of Health Sciences, Etlik Zübeyde Hanım Women’s Health Training and Research Hospital, Ankara Turkey

**Keywords:** Hysterectomy, ovarian reserve, rat, Doppler ultrasonography, PPAR gamma, rosiglitazone

## Abstract

**Background/aim:**

To evaluate the effects of hysterectomy on rat ovaries and the possible protective role of peroxisome proliferator-activated receptor gamma (PPAR-γ) agonist-rosiglitazone against ovarian reserve decrement.

**Materials and methods:**

Forty-five adult Wistar albino rats were randomly divided into three groups. Hysterectomy was performed (n = 15) in group 1 [H]; 1 mg/kg/day PPAR-γ agonist/rosiglitazone was used for 50 days after hysterectomy (n = 15) in group 2 [H + R]; a sham operation was performed (n = 15) in group 3 [control, C]. Blood samples were collected for anti-Müllerian hormone (AMH) evaluation in all groups and simultaneous ovarian Doppler examination was performed in [H] and [H + R] groups before and after (50 days) hysterectomy. All animals were sacrificed to obtain ovaries for histological examination.

**Results:**

AMH levels were found to be significantly decreased at postoperative day 50 in all groups (P < 0.05). Histopathologic analysis showed that primary, preantral, and antral follicle counts were significantly higher in the [H] group as compared to the [C] and [H + R] groups (P < 0.05). There was no significant difference between the [C] and [H + R] groups in terms of follicle numbers (P > 0.05). In the ovarian Doppler blood flow analysis, all parameters were significantly decreased in group [H] (P < 0.05), but not in the [H + R] group (P > 0.05) on postoperative day 50.

**Conclusion:**

Hysterectomy affects the histopathological structure of rat ovaries and PPAR-γ agonist-rosiglitazone improves the ovarian Doppler blood flow parameters.

## 1. Introduction

Hysterectomy with ovarian preservation is widely used for treating a variety of gynecologic conditions such as symptomatic uterine fibroids, abnormal uterine bleeding, chronic pelvic pain, etc. in premenopausal women. Many studies have investigated the possible effects of hysterectomy on ovarian reserve, ovarian blood flow, and ovarian histology. Previously, it has been considered that the ovaries, which are close to the uterus, are affected by hysterectomy [1–5]. Although it is clear that most women do not lose ovarian function in the short-term after hysterectomy, there has long been a suspicion that these women are at an increased risk of early decrement in ovarian reserve [6]. Changes in endocrine function may impact the age-associated decline in physical function (activities including bathing, dressing, walking, carrying groceries, etc. according to the physical function subscale of the Medical Outcomes Study Short-Form 36) [7] and these changes may be accelerated by hysterectomy. Also, earlier menopause, in turn, has serious health implications, including an increased risk of osteoporosis, cardiovascular disease, and all-cause mortality [8,9]. However, currently, there are no known treatment options to help prevent the possible damage that may occur in the ovaries after hysterectomy.

In vitro/in vivo studies on the use of the peroxisome proliferator-activated receptor-γ (PPAR-γ) agonist rosiglitazone as an antidiabetic agent have previously shown that this agent has antiapoptotic, antihypoxia, antioxidant, antiinflammatory, neovascularization, and antiproliferative effects [10,11]. PPAR-γ receptors provide folliculogenesis, luteal phase, and ovulation control with effects on tissue repair [12]. Also, the stimulation of PPAR-γ receptors provides for the expression of vascular endothelial growth factor (VEGF), which is responsible for the formation of new vessels in ovaries [13]. In addition to these effects on angiogenesis, PPAR-γ causes an increase in ovarian vascularization by the regulation of endothelin-1 (ET-1) and nitric oxide synthetase (NOS) [13]. 

In the present study, we used a female rat model that allowed for ovarian histopathologic assessment, with the aim of determining the possible effects of hysterectomy on ovaries. We planned to research the effect of hysterectomy on the ovaries by examining anti-Müllerian hormone (AMH) levels and ovarian Doppler blood flow, and by engaging in histopathologic evaluation. Additionally, we attempted to investigate the validity of the hypothesis that PPAR-γ agonist-rosiglitazone can prevent the undesired effects that may occur in the ovaries after hysterectomy.

## 2. Materials and methods

### 2.1. Animals

Forty-five sexually mature, adult, cycling, nonpregnant Wistar albino rats weighing between 200–250 g (2–3 months old) were used in this study. The rats were housed in individual metabolic cages for 10 days to acclimatize them to the study surroundings. Throughout the experiment, environmental conditions were kept constant: 12 L/12 D cycle (lights off at 18:00 h), room temperature of 24 ± 1 °C, and 55% ± 5% relative humidity. The study was approved by the ethical committee of Ankara Education and Research Hospital, Ankara, Turkey (33/0001-29.01.2011) and conducted at the same hospital’s Animal Research Center (May–August 2011). All investigations complied with the 1996 National Academy of Science’s Guide for the Care and Use of Laboratory Animals. 

### 2.2. Study design

**Figure 1:**
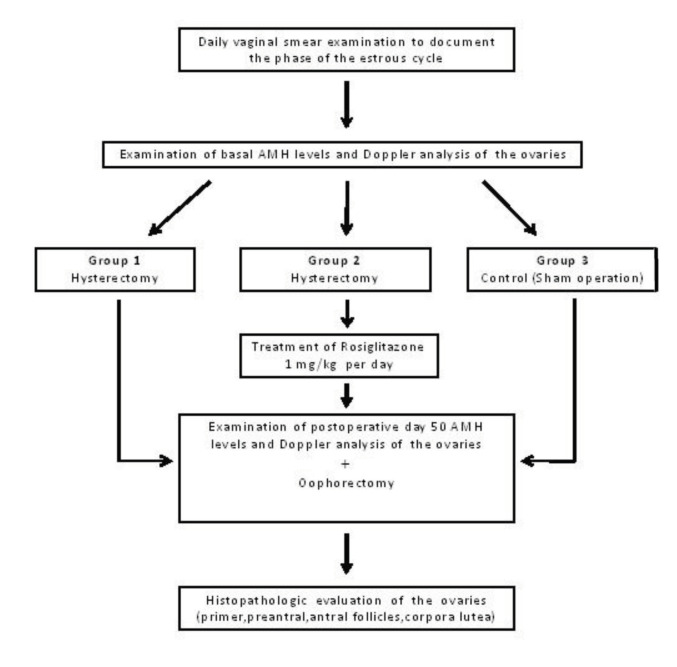
Flow chart of the study.

Forty-five nonpregnant adult female Wistar albino rats were used in this study. One week after acclimation, vaginal smears were performed daily to document the phase of the estrous cycle. After 2 complete estrous cycles, the rats in the estrous phase were selected and randomized into the 3 groups. In group 1 (n = 15), only hysterectomy was performed [H]; group 2 received rosiglitazone maleate (Avandia, GlaxoSmithKline, Istanbul, Turkey) for 50 days after hysterectomy (n = 15) [H + R]; group 3 (n = 15) served as a control group [C] and underwent a sham operation. Hysterectomy was performed in the study groups ([H] and [H + R]) by the same technician (OLT) according to the technique described by Waynforth and Flecknell [14,15]. Basal and day-50 AMH levels were measured in all groups. Doppler examination was performed in the [H] and [H + R] groups to evaluate the ovarian blood flow at basal and postoperative day 50 during the operations. At postoperative day 50, oophorectomy was performed on the rats in the estrous phase, confirmed by vaginal smear. Then, all rats were sacrificed by decapitation. Basal and postoperative day 50 AMH levels, Doppler analysis, and ovarian histopathology were compared within and between the groups (Figure 1).

With this study design, we tried to answer the following questions as our study outcomes: (1) does hysterectomy affect the ovaries? and (2) can PPAR-γ agonist-rosiglitazone therapy counteract the possible effects of hysterectomy on the ovaries?

### 2.3. Vaginal smears

Vaginal smears were performed daily to document the phase of the estrous cycle. Five phases of the estrous cycle have previously been specified in the literature [14,15]. Homogenization was ensured by operating on the rats in the estrous phase, which was determined through daily vaginal smear samples.

### 2.4. Hysterectomy

**Figure 2:**
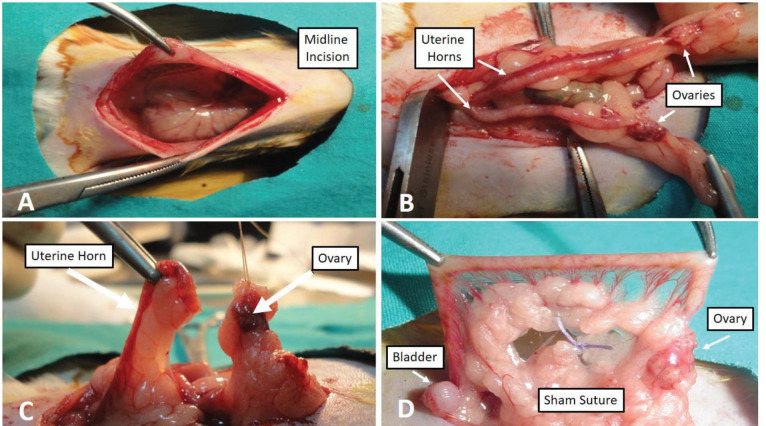
(2a). Entry to the abdomen with a midline incision. (2b). Appearance of uterine horns and ovaries taken out of the abdomen. (2c). Ovary separated from uterine horn. (2d). Sham suture placed on fatty tissue so as not to disrupt ovarian blood supply.

A mixture of ketamine, xylazine, and acepromazine (150:30:5 mg/mL) anesthetics was administered at a dose of 0.6 mL/kg to rats in the estrous phase. Afterward, while in the supine position, the abdominal skin of the rats was shaved prior to surgery and a 10% povidone-iodine solution was applied to ensure antisepsis. In compliance with sterility conditions, the abdomen was entered with a midline incision starting from the top of the urethral orifice and extending to 3-quarters of the abdomen. Each uterine horn was pulled out and laid on warm moistened gauze. The utero-ovarian artery and vein were ligated with a single suture, both anteriorly and posteriorly, while the collateral supply to the ovaries and cervix was preserved. Each uterine horn was cut at its junction with the fallopian tube and transected at the cervix. Then, the abdomen was closed by suturing in layers [14,15] (Figure 2a–2d). Subsequently, the rats were left to recover.

### 2.5. Rosiglitazone treatment

Rosiglitazone maleate 1 mg/kg per day was given to the [H + R] group starting on the day of the surgery and continuing for 50 days (n = 15) [10]. The drug dose was calculated according to the rats’ body weight. The rosiglitazone was given via orogastric tubes after being dissolved in 2 mL of saline. Every day, each rat was held at the back of the neck region by an experienced technician. The head was gently squeezed and fixed with the help of the thumb and the index finger and an orogastric tube were inserted into the stomach [11]. We have not seen any side effects with rosiglitazone.

### 2.6. Measurement of ovarian Doppler blood flow

After entry into the abdomen of the rats in the [H] and [H + R] groups, following the visualization of uterine horns, the uterine horns were removed and the right and left ovaries were seen. All measurements were taken by the same person (SBM) using a portable LOGIQ Book XP ultrasound (General Electric Healthcare, Solingen, Germany), with a 6–10 MHz linear probe in a manner that had a minimal mechanical effect on the ovaries [16]. At least 3 Power Doppler US (angio mod) images were taken for each ovary. Images with suitable quality were stored in the DICOM for Power Doppler. Afterward, the Pixelflux program (Version 1.0, Chameleon-Software, Leipzig, Germany) was utilized to obtain dynamic tissue perfusion measurements on the Power Doppler images. The temporal variation in intensity (I) red, Vred, average intensity, and whole intensity values were statistically evaluated [16].

### 2.7. Measurement of hormonal parameters

Under the influence of anesthesia, 1–1.5 mL of blood samples were directly taken intracardially at basal and postoperative day 50 before decapitation. The collected blood was centrifuged and the obtained serum was stored at –20 °C for 55 days for basal samples and 5 days for postoperative day-50 samples. AMH levels were measured preoperatively upon enrollment and at postoperative day 50. Serum AMH levels were measured by enzyme-linked immunosorbent assay (ELISA) using a commercially available rat AMH ELISA kit (E90228RA, USCN Life Science Inc., Cloud-Clone Corp., Houston, TX, USA). The mean sensitivity of the assays was <0.084 ng/mL.

### 2.8. Histopathologic evaluation of the ovaries

The ovaries of each group were excised at postoperative day 50 after Doppler analysis and before blood sampling and decapitation. They were cleared of all adhering fat, weighed, and processed for histology upon completion of the studies. Tissues fixed in neutral formaldehyde were washed, trimmed, dehydrated in a graded series of alcohol, and cleared in xylene. The tissues were embedded in paraffin wax and blocks were prepared. Ovarian sections (6 µm) were obtained from both groups and stained with hematoxylin and eosin (H&E). Afterward, through the use of a light microscope, the number of primer, preantral, and antral follicles and corpora lutea was determined by simple point counting on serially sectioned left and right ovaries. All procedures were performed by the same pathologist (KHM).

### 2.9. Statistical analyses

Statistical Package for the Social Sciences 20.0 (SPSS Inc., Chicago, IL, USA) was used to record and statistically analyze the data. Normality was tested by the Shapiro–Wilk test. Nonnormally distributed metric variables were analyzed by the Kruskal–Wallis test for comparison of variables among groups. If the difference was significant, the binary comparisons were done with the Mann–Whitney U test. The variables in the same group (basal and postoperative day 50) were analyzed by Wilcoxon’s signed-rank test. Values were expressed as mean ± standard deviation (SD) and median (min–max), and statistical significance was defined as P < 0.05.

## 3. Results

One rat in the [H] group and 1 rat in the [H + R] group were lost in the follow-up period. The study was completed with the remaining 43 rats for all groups.

Baseline serum AMH values were similar for all groups (P > 0.05). Serum AMH levels significantly decreased in all 3 groups on postoperative day 50 (P < 0.05, P < 0.05, P < 0.01; P values for the [H], [H + R], and [C] groups, respectively), but there was no significant difference between the groups (P > 0.05) (Table 1).

**Table 1:** Comparison of basal and postoperative 50th-day AMH.

AMH	[H] group (n = 14) Median (min–max)	[H + R] group (n = 14) Median (min–max)	[C] group (n = 15) Median (min–max)	P value^a^
Basal AMH	0.72 (0.68–0.79)	0.72 (0.68–7.30)	0.78 (0.65–7.75)	>0.05
50th day AMH	0.62 (0.37–6.30)	0.65 (0.59–0.79)	0.64 (0.58–0.70)	>0.05
P value^b^	<0.05*	<0.05*	<0.01*	

AMH: anti-Müllerian hormone; [H]: hysterectomy; [H + R]: hysterectomy + rosiglitazone; [C]: control; min–max: minimum–maximum. ^a^ P value comparing basal and postoperative 50th-day AMH levels among groups. ^b^ P value comparing basal and postoperative 50th-day AMH levels within groups. * statistically significant.

**Figure 3:**
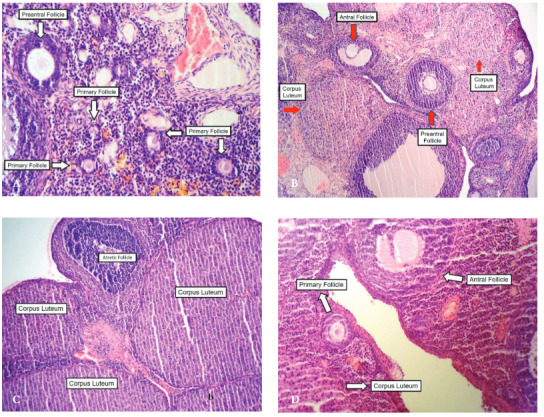
(3a). Section of an ovary from the [H] group showing numerous primary follicles, and preantral follicle [×200, (H&E)]. (3b). Section of an ovary from the [H + R] group showing preantral and antral follicles and corpus luteum [×100, (H&E)]. (3c). Section of an ovary from the [H + R] group showing corpus luteum and atretic follicle [×200, (H&E)]. (3d). Section of an ovary from the [C] group showing primary and antral follicles, and corpus luteum [×200, (H&E)].

When the ovaries were evaluated histopathologically, the primary, preantral, and antral follicle numbers in the [H] group were determined to be higher than those of the [H + R] and [C] groups (P < 0.001, P < 0.01, P < 0.05; P values for primary, preantral, and antral follicles, respectively) (Table 2). However, corpora lutea numbers were found to be similar between the groups (P > 0.05) (Figures 3a–3d). 

**Table 2:** Comparison of the histopathologic examination of the ovaries between the groups.

	[H] group (n = 14) Median (min–max)	[H + R] group (n = 14) Median (min–max)	[C] group (n = 15) Median (min–max)	P values^a^
Primary follicles	7.5 (3.0–14.0)	2.0 (0–9.0)	3.0 (1.0–5.0)	<0.001*
Preantral follicles	8.0 (3.0–14.0)	4.0 (2.0–11.0)	4.0 (2.0–7.0)	<0.01*
Antral follicles	11.0 (3.0–18.0)	5.5 (3.0–13.0)	7.0 (4.0–12.0)	<0.05*
Corpora lutea	14.0 (8.0–36.0)	11.5 (0–20.0)	12.0 (4.0–28.0)	>0.05
	[H] vs. [H + R] P value^b^	[H] vs. [C] P value^c^	[H + R] vs. [C] P value^d^	
Primary follicles	<0.01*	<0.01*	>0.05	
Preantral follicles	<0.01*	<0.01*	>0.05	
Antral follicles	<0.05*	<0.05*	>0.05	
Corpora lutea	>0.05	>0.05	>0.05	

[H]: hysterectomy; [H + R]: hysterectomy + rosiglitazone; [C]: control; min–max: minimum–maximum. ^a^P value comparing follicle numbers among groups. ^b^P value comparing follicle numbers of [H] vs. [H + R]. ^c^P value comparing follicle numbers of [H] vs. [C]. ^d^P value comparing follicle numbers of [H + R] vs. [C].

When the ovarian Doppler blood flow results were assessed on postoperative day 50, a significant decrease was observed in the [H] group for all parameters [intensity (I)-red average, Vred average, whole intensity, average intensity], while there was no decrement for all of these parameters in the [H + R] group (P > 0.05) (Table 3). 

**Table 3:** Comparison of basal and postoperative 50th-day Doppler blood flow results between [H] and [H + R] groups.

Doppler parameters	Groups	Basal Mean ± SD (min–max)	50th Day Mean ± SD (min–max)	Difference of 2 days Median (min–max)	P value^a^
Ired average	[H]	0.14 ± 0.1 (0.04–0.035)	0.07 ± 0.04 (0.02–0.15)	0.04 (–0.07–0.30)	<0.05*
	[H + R]	0.09 ± 0.1 (0.02–0.21)	0.13 ± 0.11 (0.02–0.37)	–0.005 (0.29–0.08)	>0.05
P value^b^		>0.05	<0.05*	<0.05*	
Vred average	[H]	0.49 ± 0.07 (0.41–0.68)	0.41 ± 0.04 (0.33–0.49)	0.07 (–0.02–0.27)	<0.01*
	[H + R]	0.47 ± 0.11 (0.38–0.84)	0.45 ± 0.11 (0.34-0.71)	0.03 (–0.23–0.47)	>0.05
P value^b^		>0.05	>0.05	>0.05	
Whole intensity	[H]	0.14 ± 0.1 (0.04–0.35)	0.07 ± 0.04 (0.02–0.15)	0.04(–0.07–0.30)	<0.05*
	[H + R]	0.09 ± 0.1 (0.02–0.21)	0.13 ± 0.11 (0.02–0.37)	–0.005 (0.29–0.08)	>0.05
P value^b^		>0.05	<0.05*	<0.05*	
Average intensity	[H]	0.07 ± 0.05 (0.02–0.18)	0.04 ± 0.02 (0.01–0.08)	0.02 (–0.04–0.15)	<0.05*
	[H + R]	0.05 ± 0.03 (0.01–0.10)	0.07 ± 0.05 (0.01–0.19)	–0.003 (0.15–0.04)	>0.05
P value^b^		>0.05	<0.05*	<0.05*	

[H]: hysterectomy; [H + R]: hysterectomy + rosiglitazone; [C]: control; min–max: minimum–maximum. ^a^P value comparison of basal and postoperative 50th-day Doppler blood flow results within [H] and [H + R]. ^b^P value comparison of basal and postoperative 50th-day Doppler blood flow results between [H] and [H + R] groups. * statistically significant.

## 4. Discussion

Our primary objective in this study was to investigate the possible effects of hysterectomy on the ovaries. In our study, we found that hysterectomy affects the ovaries, and PPAR-γ agonist-rosiglitazone therapy improves the ovarian Doppler blood flow parameters in rats.

There are some studies on the measurement of serum AMH concentrations in women who have had a hysterectomy with ovarian conservation. The study by Lee et al. measured serum AMH levels at the baseline, 1st week, 1st month, and 3rd month after hysterectomy; no significant changes were found in AMH levels [17]. Hehenkamp et al. measured serum FSH and AMH levels in patients in whom uterine artery ligation and hysterectomy had been performed. In that study, mean AMH levels were significantly decreased at the postoperative 6th week and had subsequently recovered to normal values between the 6th week and the 12th month; they were found to have remained stable at the 24-month follow-up. As a result, it was reported that hysterectomy and uterine artery ligation might affect the ovarian reserve [18]. Wang et al. measured AMH levels following hysterectomy and found that serum AMH levels were significantly lower 2 days and 3 months following hysterectomy compared to the preoperative level [19]. In another study, Atabekoglu et al. measured preoperative and postoperative 4th-month serum AMH levels in premenopausal women who underwent hysterectomy; they found no significant change [20]. In that study, serum AMH levels decreased at the 4th month for hysterectomy patients and controls, which is similar to our results [20]. In our study, AMH levels were significantly decreased in both the hysterectomy and control groups over time; however, even if the decrease of AMH levels in the hysterectomy group was accounted for by the ovaries being affected by hysterectomy, it is difficult to explain the decrease in the control group with a similar mechanism. This situation may be explained by the dissimilarity of AMH metabolism in humans and rodents and also may be associated with surgical stress, anesthetic substances, the postoperative process, and potential changes in the periovarian microenvironment – factors that may affect the AMH levels of rats in the control group [21–23]. By contrast, if we had a longer follow-up period, similar to Hehenkamp’s study [18], AMH levels may have shown fluctuations and reached their normal values in the following period. Furthermore, genetic variation in AMH and AMHR type II (AMHRII) receptor genes were shown in other studies and could be another explanation for our results [24]. 

Morphological evaluation of the ovaries may allow for a more definite understanding of the effects of hysterectomy; however, this type of evaluation is not easy in humans. Sauza et al. reported the potential long-term effects of hysterectomy on ovarian morphology for the first time [25]. They performed bilateral ovarian biopsies; then, 1 year after hysterectomy, they performed a laparoscopy and repeated the ovarian biopsy. They reported that the ovarian histology showed an impressive reduction in the follicular reserve, in addition to the thickening of the tunica albuginea and stromal hyperplasia. It was also reported that the total number of ovarian follicles in women with hysterectomy appeared to be significantly lower than the number of follicles before the operation [25]. Finally, they concluded that hysterectomy negatively influenced ovarian function in terms of histology. Ozdamar et al. evaluated ovarian function in rats following hysterectomy and found that the numbers of preantral and antral follicles decreased, while the numbers of atretic follicles and corpus luteum increased significantly 6 months after hysterectomy [26]. In the study conducted by Tapisiz et al. on rats, when the ovaries were assessed histopathologically on the 100th day after hysterectomy, a decrease in primary, preantral, and antral follicle numbers was reported [15]. The follow-up period in our study is shorter than those in the other studies, and unlike previous studies, we found a significant increase in primary, preantral, and antral follicle numbers in the hysterectomy group on posthysterectomy day 50. Similarly, Tanaka et al. demonstrated that hysterectomy increased the number of ovulations in rats [27]. This condition may be explained by the stimulation of the hypothalamo-hypophyseal axis and the start of an autocatalytic cycle as a result of the removal of the uterus’s inhibitory effect on follicular development [27]. If the follow-up period had been longer, such as in the study of Ozdamar et al. and Tapisiz et al., we may have observed a decrease in the number of follicles based on diminishing ovarian reserve [15,26]. Perhaps our study could show the short-term effects of hysterectomy on ovarian morphology. Another point to be considered is the contradiction between the increased number of follicles and the decreased levels of AMH produced by the follicle and an indirect evaluation of the follicle pool. Pellatt et al. examined the production of AMH from follicles and compared AMH levels between ovulatory and anovulatory patients with polycystic ovary (PCO) syndrome. At the end of the study, they found that AMH production per granulosa cell was 75 times higher in anovulatory PCOs as compared to normal ovaries and concluded that the raised levels of AMH could be misleading if used as a marker of ovarian reserve [28]. In addition, it has been reported that some biological (race, ethnicity, polymorphisms of AMH and its receptor, systemic illness, etc.), reproductive (parity, endometriosis, ovarian suppression, etc.), and environmental/lifestyle (body mass index, current smoking, low vitamin-D level, etc.) factors may affect AMH levels [29,30]. Consequently, AMH levels may not always clearly reflect the ovarian reserve.

The other controversial issues that are thought to cause a decrease in posthysterectomy ovarian reserve are the changes occurring in ovarian blood flow [31–34]. Janson et al. demonstrated an acute reduction of ovarian blood flow immediately after ligation of the uterine vessels by using 133Xenon; they assumed that this reduction in blood flow was responsible for the “steroid drop” after hysterectomy [34]. Chan et al. found that the ovarian stromal blood flow was significantly reduced in women with a hysterectomy and demonstrated that ovarian functions might be changed [31]. Recently, Lee et al. did not find a significant difference between the blood flow rate measures of ovarian arteries before hysterectomy and after a 3-month follow-up period, which is inconsistent with others [17]. Our study is the first animal model that examines the effects of hysterectomy via Doppler ultrasonography on ovarian blood flow. We found a significant decrease in ovarian blood flow on postoperative day 50 in the hysterectomy group. Based on the current results, it may be said that hysterectomy may disrupt the ovarian blood supply. It has also been reported that hysterectomy affects the hemodynamics of ovarian circulation resulting from the intervention. Through ligation of the uterine artery during the hysterectomy, the ovarian arteriole pressure increases, leading to vascular hypertension followed by constriction of arterioles; this results in a progressive decrease in ovarian circulation and consequent ovarian tissue damage [34,35].

The secondary objective of our study was to investigate the effects of PPAR-γ agonist rosiglitazone as an antioxidant, antiinflammatory therapeutic agent on the ovaries after hysterectomy. PPAR-γ receptor is a ligand-dependent transcription factor and a member of the nuclear receptor superfamily. Acting as sensors of hormones, vitamins, endogenous metabolites, and xenobiotic compounds, the nuclear receptors control the expression of a very large number of genes [10–13,36]. Although the literature contains many publications that indicate the antioxidant and antiinflammatory effects of PPAR-γ agonists on other organs [8,9], there is no study about its potential effects on posthysterectomy ovaries. Our study has the distinction of being the first animal model performed in connection with this subject. We found a significant increase in ovarian blood flow in the rosiglitazone group. Based on our results and those of other studies [13,36], it may be speculated that rosiglitazone increases ovarian blood flow after hysterectomy. Additionally, the amount of follicular increase in the hysterectomy group was not seen in the rosiglitazone group. Rosiglitazone may inhibit increased ovulation as a result of the autocatalytic cycle that may occur after the hysterectomy, described by Tanaka et al. [27]. These findings may indicate the protective effect of rosiglitazone. 

Several limitations of this study must be acknowledged. The first limitation was the shortness of the follow-up period. However, the estrous cycle of rats was 4–5 days, and in a 50-day follow-up period, a length of 10–12 cycles, was considered to be analogous to a similar one-year follow-up period in humans. Multiovulation in rats may not be generalized to ovulation in humans, which is the second limitation. It should also be noted that the study results may contain both type 1 and type 2 errors. Furthermore, the limited number of rats in each group, due to ethical principles, was a limitation that may weaken the statistical significance of these studies. Last but not least, the difficulty involved in adapting the results of our study, an animal model, to clinical application in humans should not be overlooked. 

In conclusion, based on the histopathologic changes and the decrease in AMH levels and ovarian blood flow findings in our study, it can be said that hysterectomy affects the ovaries. Additionally, the use of PPAR-γ agonist-rosiglitazone may reduce the potential negative effects of hysterectomy on ovaries. Further studies with larger numbers of human participants are needed to reach more definite conclusions.

## Acknowledgment

The authors wish to thank Kenan Köse for his helpful comments and suggestions about statistical analysis. In addition, we thank the veterinarian Cengiz Yalçın, the director of Ankara Education and Research Hospital’s Animal Research Center, and the lab staff Erkan Kaya and Emir Kuşcu for helping at every stage of the experiment. The authors declare that there is no conflict of interest. This experimental study was approved by the ethical committee of Ankara Education and Research Hospital, Ankara, Turkey [33/0001-29.01.2011].
